# Supplementary material for: Evolutionary dynamics of the successful expansion of pandemic Vibrio parahaemolyticus ST3 in Latin America
Source: Nat Commun. 2024 Sep 7;15:7828. doi: 10.1038/s41467-024-52159-y (PMC11380683; doi:10.1038/s41467-024-52159-y)
Supplement: Supplementary file 1 — Supplementary Information [file 41467_2024_52159_MOESM1_ESM.pdf]

Supporting Information

Evolutionary dynamics of the successful expansion of pandemic *Vibrio parahaemolyticus* ST3 in Latin America

Amy Marie Campbell[1,2], Ronnie G. Gavilan[3,4], Michel Abanto Marin[5], Chao Yang [6], Chris Hauton[1], Ronny van Aerle[2], Jaime Martinez-Urtaza[2,4*]

[1] School of Ocean and Earth Science, University of Southampton, National Oceanography Centre, Southampton, UK

[2] Centre for Environment, Fisheries and Aquaculture Science (CEFAS), Weymouth, UK

[3] Centro Nacional de Salud Pública, Instituto Nacional de Salud, Lima, Peru.

[4] Department of Genetics and Microbiology, Autonomous University of Barcelona, Barcelona, Spain

[5] Genomics and Bioinformatics Unit, Scientific and Technological Bioresource Nucleus (BIOREN), Universidad de La Frontera, Temuco, Chile.

[6] The Center for Microbes, Development and Health, CAS Key Laboratory of Molecular Virology and Immunology, Shanghai Institute of Immunity and Infection, Chinese Academy of Sciences, Shanghai, China.

Materials & Correspondence Author: *Jaime Martinez-Urtaza (jaime.martinez.urtaza@uab.cat)

This PDF file includes:

Figures S1 to S9

Tables S1 to S8

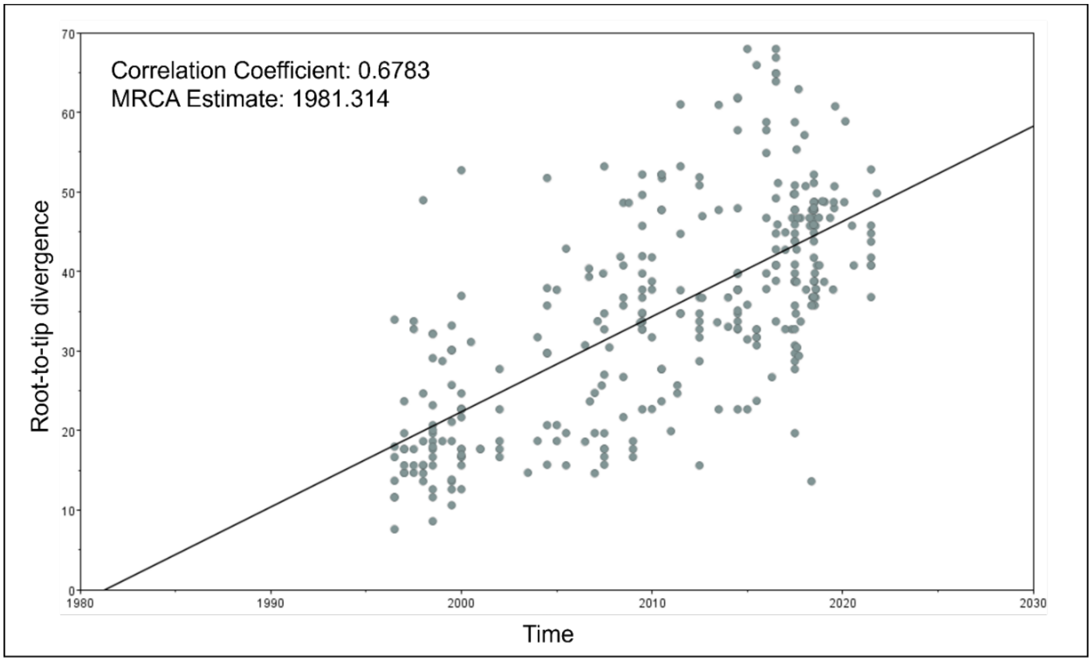

Fig. S1. Temporal signal of VpST3 collection in TempEst v1.5.3 (Rambaut et al., 2016)

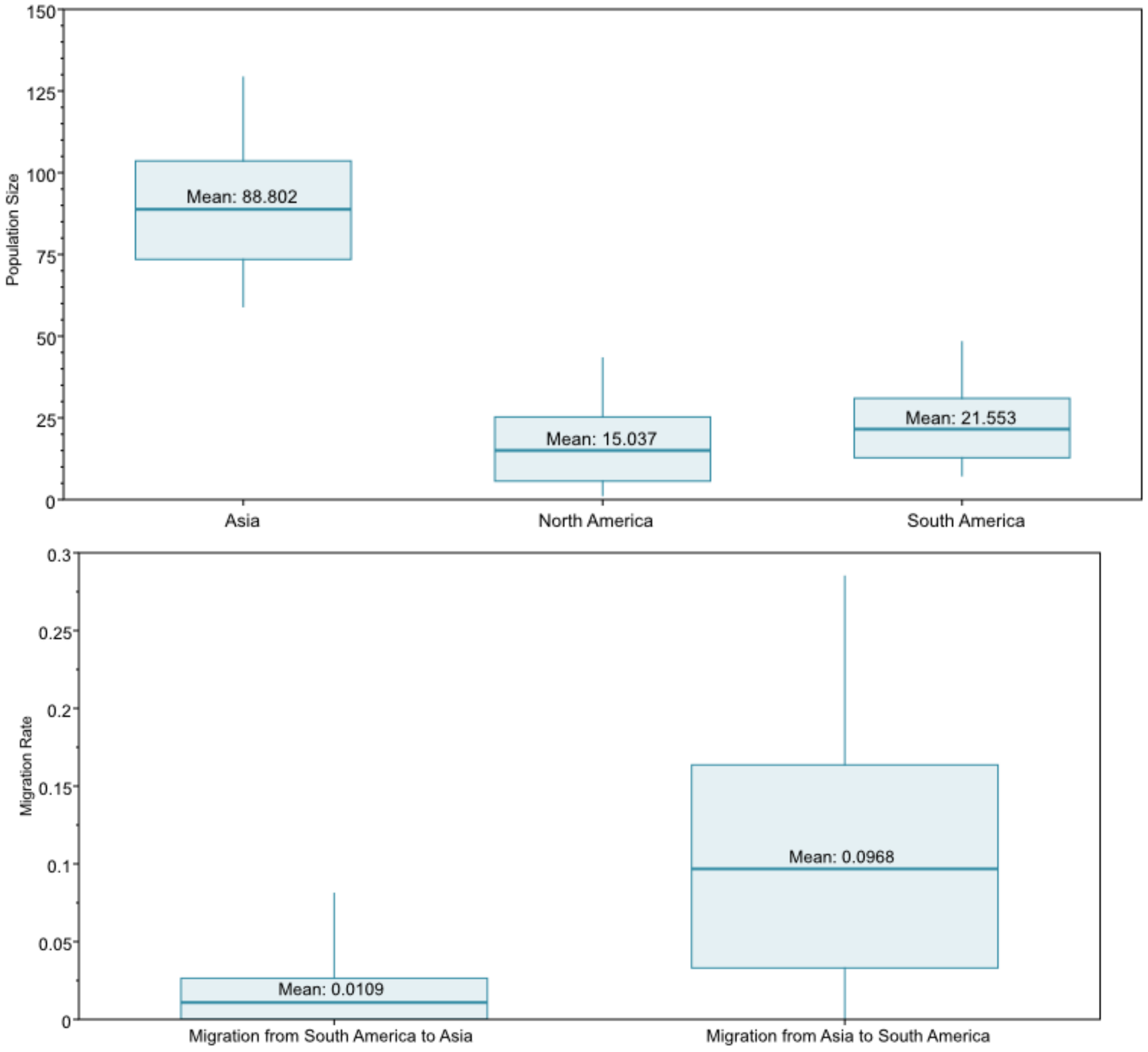

Fig. S2. Migration model from BEAST2 structured coalescent analysis. Estimated population sizes per continent (top) and estimated migration rates between South America and Asia in VpST3 collection (bottom)

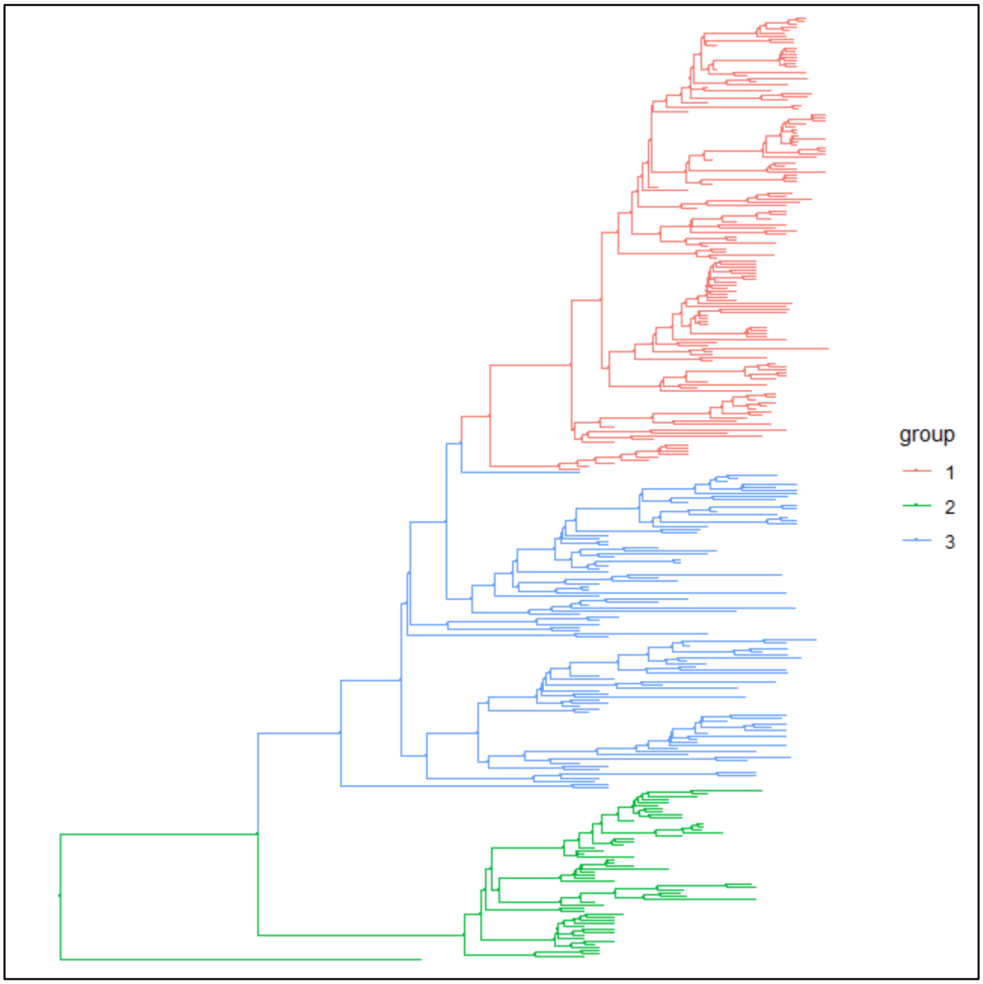

Fig. S3. Clusters identified at a significance level of p<0.0001

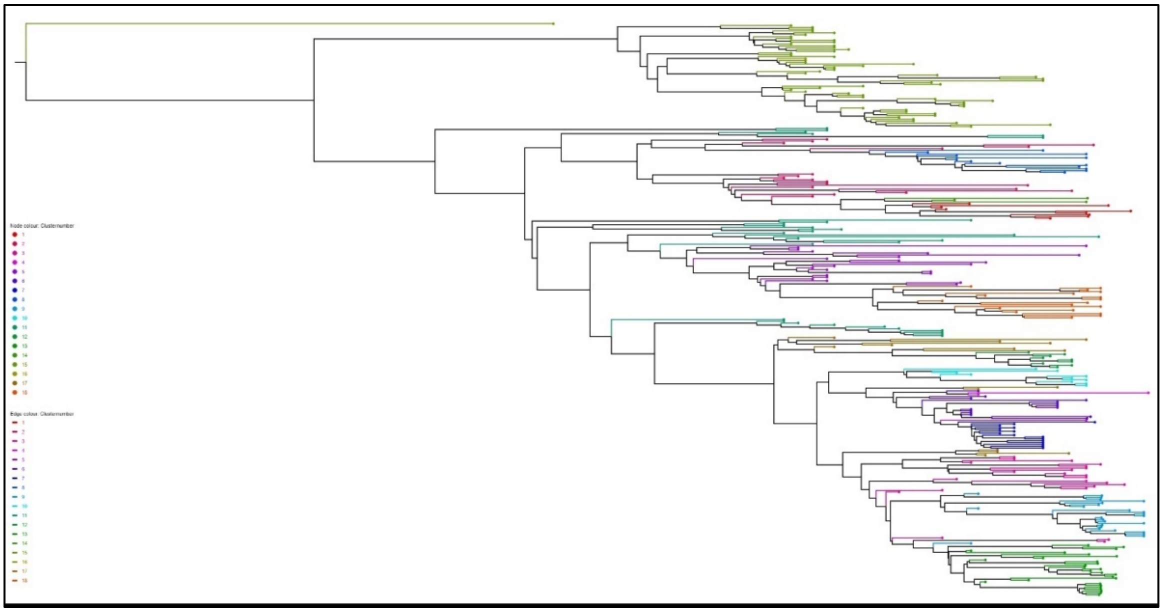

Fig. S4. Grouping of phylogenetic tree based on discriminatory analysis of principle components (DAPC). Based on natural clusters (LatAm-VpST3 is represented in a single cluster- top group in olive green)

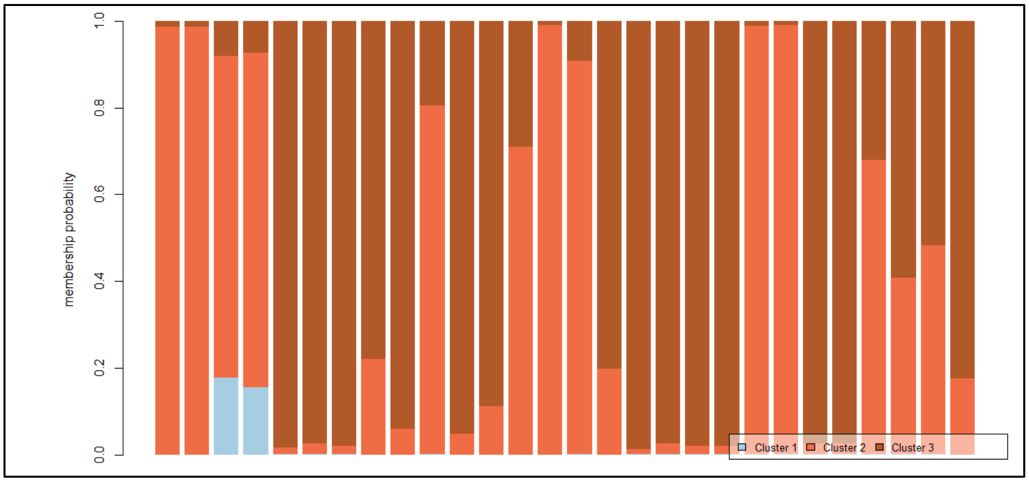

Fig. S5. Evidence of admixture within 28 individuals in the VpST3 collection, based on three identified clusters (LatAm-VpST3, represented by Cluster 1, is involved in comparatively little admixture)

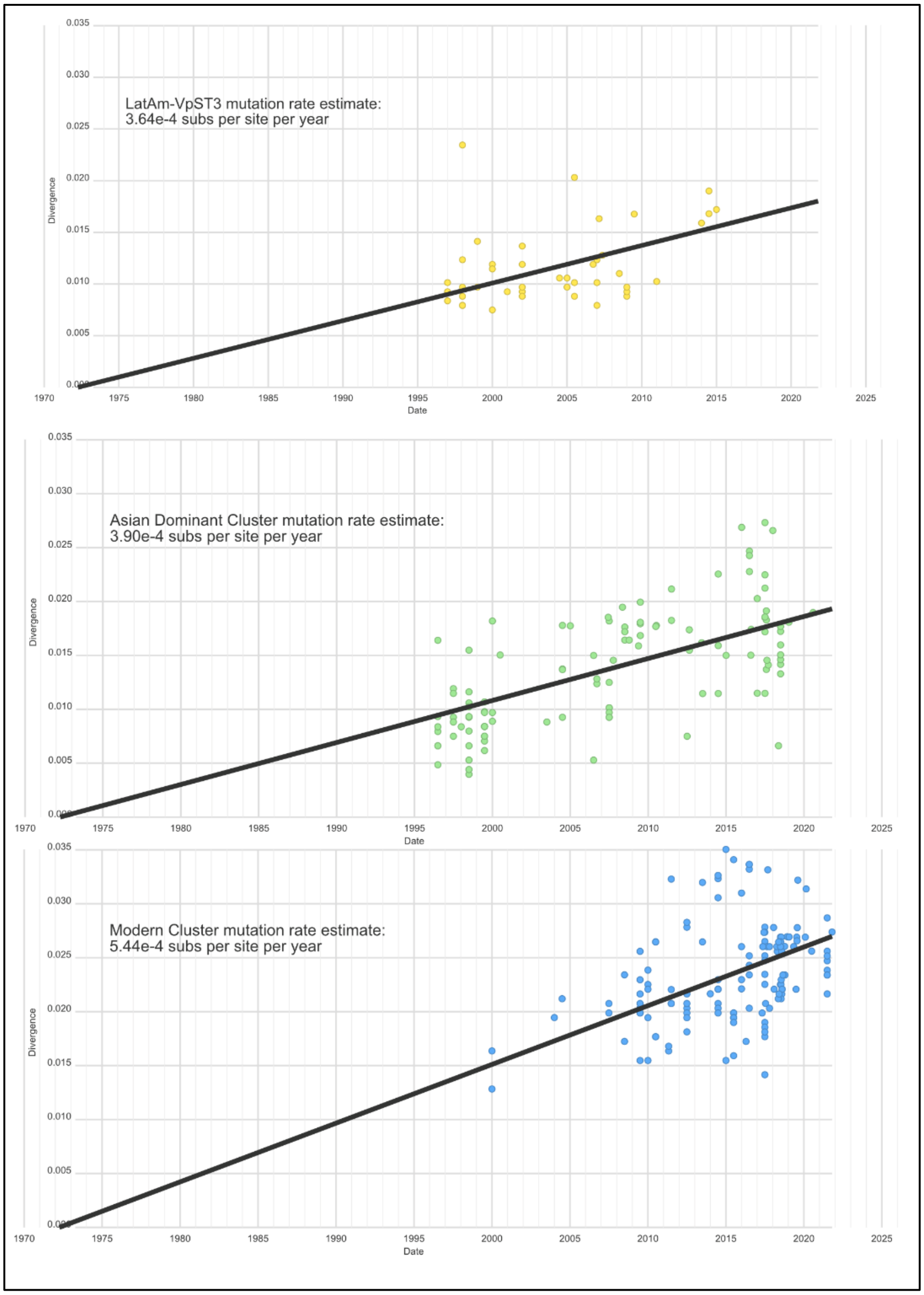

Fig. S6. Mutation rates, based on divergence, estimated for each cluster

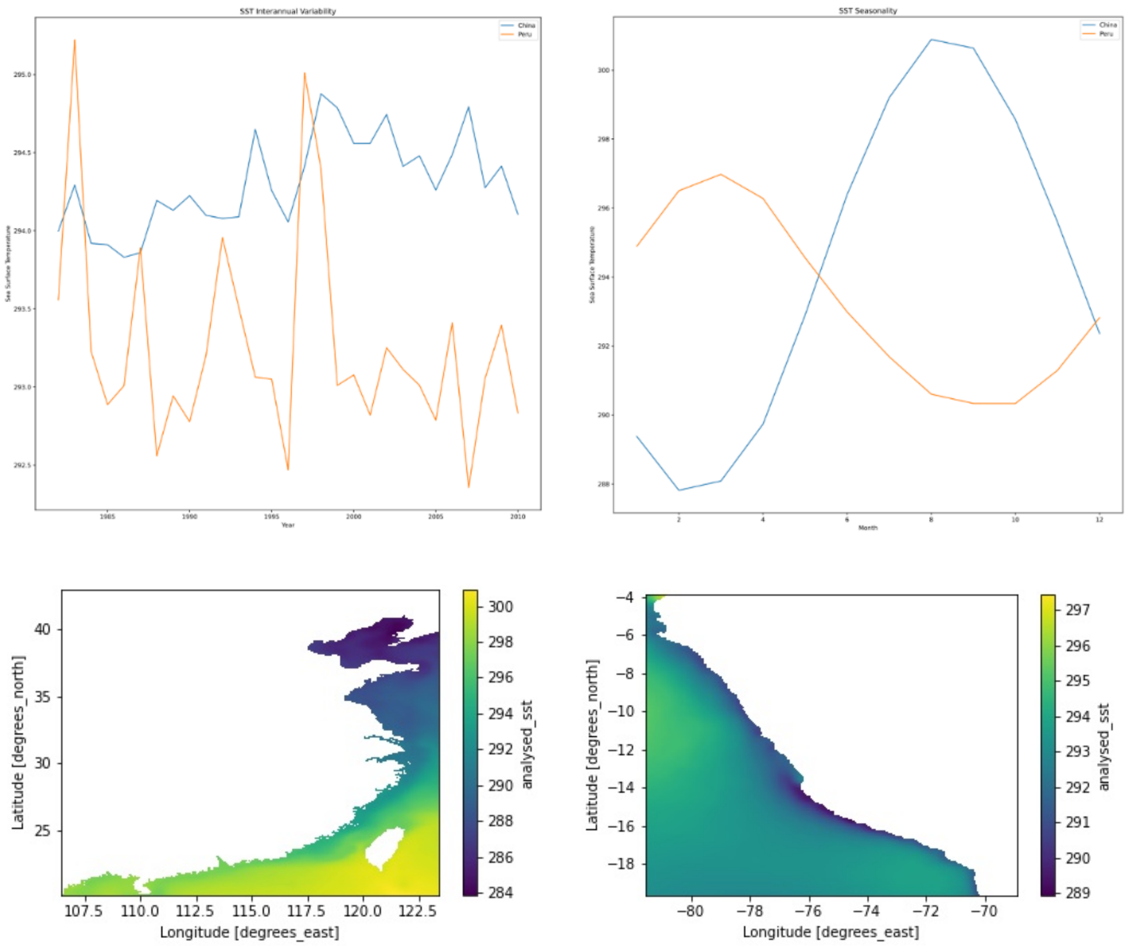

Fig. S7. Comparative climate trends (interannual variability, seasonality, per-pixel average) for sea surface temperature of coastal waters off China (bottom-left)- dominant origin country of co-existing dominant Asian group)- and Peru (bottom-right)- dominant location of LatAm-VpST3

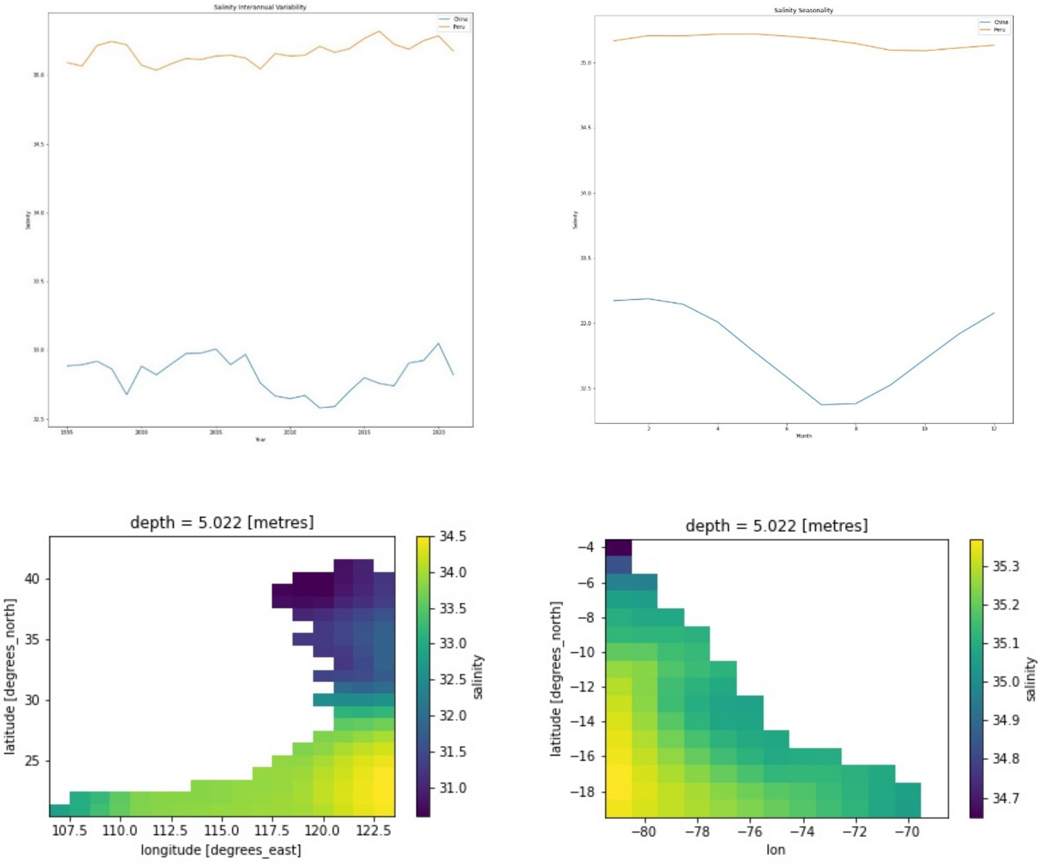

Fig. S8. Comparative climate trends (interannual variability, seasonality, per-pixel average) for salinity of coastal waters off China (bottom-left)- dominant origin country of co-existing dominant Asian group)- and Peru (bottom-right)- dominant location of LatAm-VpST3

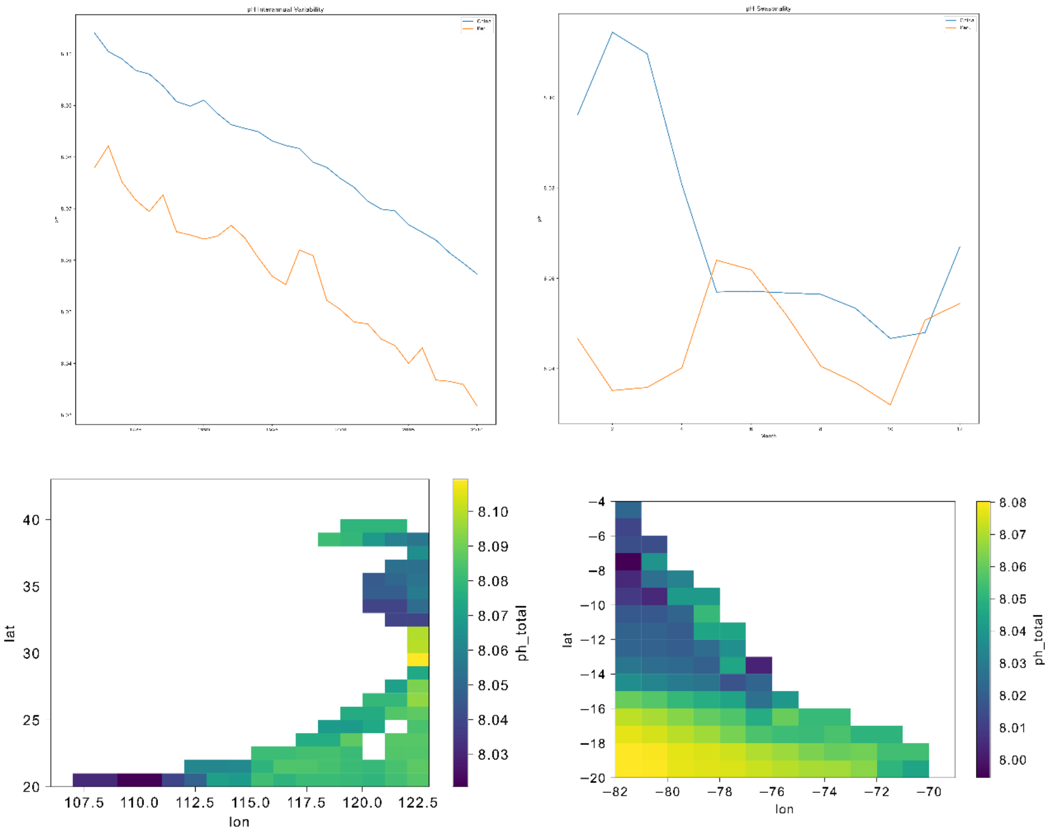

Fig. S9. Comparative climate trends (interannual variability, seasonality, per-pixel average) for pH of coastal waters off China (bottom-left)- dominant origin country of co-existing dominant Asian group)- and Peru (bottom-right)- dominant location of LatAm-VpST3

Table S1. VpST3 collection metadata

Name	Strain	Isolation type	Collection date	Continent	Country	Region	BioSample	BioProject	NewSamples
A1078098_1998-1-01_SouthAmerica_Peru	A10_780-98	NA	1998	SouthAmerica	Peru		SAMN40891081	PRJNA1062747	NewlyUsed
A11A1198_1998-1-01_SouthAmerica_Peru_Lima	784-98	clinical	1998	SouthAmerica	Peru	Lima	SAMN40891082	PRJNA1062747	NewlyUsed
A12A1298_1998-1-01_SouthAmerica_Peru_Lima	971-98	clinical	1998	SouthAmerica	Peru	Lima	SAMN40891083	PRJNA1062747	NewlyUsed
A3A397_1997-1-01_SouthAmerica_Peru_Chancay	763-97	clinical	1997	SouthAmerica	Peru	Chancay	SAMN40891084	PRJNA1062747	NewlyUsed
A4A497_1997-1-01_SouthAmerica_Peru_Cajamarca	790-97	clinical	1997	SouthAmerica	Peru	Cajamarca	SAMN40891085	PRJNA1062747	NewlyUsed
A5A597_1997-1-01_SouthAmerica_Peru_Lambayeque	875-97	clinical	1997	SouthAmerica	Peru	Lambayeque	SAMN40891086	PRJNA1062747	NewlyUsed
A6A697_1997-1-01_SouthAmerica_Peru_Lima	906-97	clinical	1997	SouthAmerica	Peru	Lima	SAMN40891087	PRJNA1062747	NewlyUsed
A8A897_1997-1-01_SouthAmerica_Peru_Lambayeque	948-97	clinical	1997	SouthAmerica	Peru	Lambayeque	SAMN40891088	PRJNA1062747	NewlyUsed
A9A997_1997-1-01_SouthAmerica_Peru_Moquegua	1171-97	clinical	1997	SouthAmerica	Peru	Moquegua	SAMN40891089	PRJNA1062747	NewlyUsed
B10B100_2000-1-01_SouthAmerica_Peru_Lima	461-00	clinical	2000	SouthAmerica	Peru	Lima	SAMN40891090	PRJNA1062747	NewlyUsed
B11B110_2000-1-01_SouthAmerica_Peru_Lima	462-00	clinical	2000	SouthAmerica	Peru	Lima	SAMN40891091	PRJNA1062747	NewlyUsed
B12B120_2000-1-01_SouthAmerica_Peru_Lima	AQ4901	clinical	2000	SouthAmerica	Peru	Lima	SAMN40891092	PRJNA1062747	NewlyUsed
B3B399_1999-1-01_SouthAmerica_Peru_Lima	276-99	clinical	1999	SouthAmerica	Peru	Lima	SAMN40891093	PRJNA1062747	NewlyUsed
B4B499_1999-1-01_SouthAmerica_Peru_Lima	278-99	clinical	1999	SouthAmerica	Peru	Lima	SAMN40891094	PRJNA1062747	NewlyUsed
B8B80_2000-1-01_SouthAmerica_Peru_Lambayeque	405-00	clinical	2000	SouthAmerica	Peru	Lambayeque	SAMN40891095	PRJNA1062747	NewlyUsed
B9B90_2000-1-01_SouthAmerica_Peru_Lima	429-00	clinical	2000	SouthAmerica	Peru	Lima	SAMN40891096	PRJNA1062747	NewlyUsed
C10C101_2001-1-01_SouthAmerica_Peru_Iquitos	572-01	clinical	2001	SouthAmerica	Peru	Iquitos	SAMN40891097	PRJNA1062747	NewlyUsed
C11C112_2002-1-01_SouthAmerica_Peru_Lima	004-02	clinical	2002	SouthAmerica	Peru	Lima	SAMN40891098	PRJNA1062747	NewlyUsed
C12C122_2002-1-01_SouthAmerica_Peru_Lima	020-02	clinical	2002	SouthAmerica	Peru	Lima	SAMN40891099	PRJNA1062747	NewlyUsed
C1C10_2000-1-01_SouthAmerica_Peru_Huaral	568-00	clinical	2000	SouthAmerica	Peru	Huaral	SAMN40891100	PRJNA1062747	NewlyUsed
C2C20_2000-1-01_SouthAmerica_Peru_Lima	706-00	clinical	2000	SouthAmerica	Peru	Lima	SAMN40891101	PRJNA1062747	NewlyUsed
C4C41_2001-1-01_SouthAmerica_Peru_Lambayeque	056-01	clinical	2001	SouthAmerica	Peru	Lambayeque	SAMN40891102	PRJNA1062747	NewlyUsed
D1D12_2002-1-01_SouthAmerica_Peru_Lima	085-02	clinical	2002	SouthAmerica	Peru	Lima	SAMN40891103	PRJNA1062747	NewlyUsed
D2D22_2002-1-01_SouthAmerica_Peru_Chiclayo	182-02	clinical	2002	SouthAmerica	Peru	Chiclayo	SAMN40891104	PRJNA1062747	NewlyUsed
D3D32_2002-1-01_SouthAmerica_Peru_Lima	240-02	clinical	2002	SouthAmerica	Peru	Lima	SAMN40891105	PRJNA1062747	NewlyUsed
F1F1_2007-1-01_SouthAmerica_Chile_PuertoMontt	PMC16.7	clinical	2007	SouthAmerica	Chile	Puerto Montt	SAMN40891106	PRJNA1062747	NewlyUsed
F2F2_1998-1-01_SouthAmerica_Chile_Antofagasta	ATC224	clinical	1998	SouthAmerica	Chile	Antofagasta	SAMN40891107	PRJNA1062747	NewlyUsed
F4F47_2007-1-01_SouthAmerica_Peru_	859-07	NA	2007	SouthAmerica	Peru		SAMN40891108	PRJNA1062747	NewlyUsed
F7F7_2007-1-01_SouthAmerica_Chile_PuertoMontt	PMC73.7	clinical	2007	SouthAmerica	Chile	Puerto Montt	SAMN40891109	PRJNA1062747	NewlyUsed
G1G1_2005-1-01_SouthAmerica_Chile_PuertoMontt	PMC39.5	clinical	2005	SouthAmerica	Chile	Puerto Montt	SAMN40891110	PRJNA1062747	NewlyUsed
G2G2_1998-1-01_SouthAmerica_Chile_Antofagasta	ATC210	clinical	1998	SouthAmerica	Chile	Antofagasta	SAMN40891111	PRJNA1062747	NewlyUsed
G3G3_1998-1-01_SouthAmerica_Peru_Lima	974-98	clinical	1998	SouthAmerica	Peru	Lima	SAMN40891112	PRJNA1062747	NewlyUsed
ABS24L001_2016-1-01_SouthAmerica_Colombia	CEFAS0023	clinical_travel	2016	SouthAmerica	Colombia		SAMN12254049	PRJNA438219	Submitted2019
BS2L001_2009-7-01_Asia_Vietnam	CEFAS0002	clinical_travel	2009	Asia	Vietnam		SAMN12254028	PRJNA438219	Submitted2019
CS3L001_2009-7-01_Asia_Thailand	CEFAS0003	clinical_travel	2009	Asia	Thailand		SAMN12254029	PRJNA438219	Submitted2019
DS4L001_2013-7-01_Asia_Thailand	CEFAS0004	clinical_travel	2013	Asia	Thailand		SAMN12254030	PRJNA438219	Submitted2019
FS6L001_2010-7-01_Asia_Thailand	CEFAS0006	clinical_travel	2010	Asia	Thailand		SAMN12254032	PRJNA438219	Submitted2019
HS8L001_2014-1-01_NorthAmerica_Cuba	CEFAS0008	clinical_travel	2014	NorthAmerica	Cuba		SAMN12254034	PRJNA438219	Submitted2019
KS10L001_2014-7-01_Asia_Thailand	CEFAS0010	clinical_travel	2014	Asia	Thailand		SAMN12254036	PRJNA438219	Submitted2019
LS11L001_2014-7-01_Asia_Thailand	CEFAS0011	clinical_travel	2014	Asia	Thailand		SAMN12254037	PRJNA438219	Submitted2019
SS15L001_2011-7-01_Asia_Thailand	CEFAS0015	clinical_travel	2011	Asia	Thailand		SAMN12254041	PRJNA438219	Submitted2019
VS18L001_2016-7-01_Asia_Thailand	CEFAS0017	clinical_travel	2016	Asia	Thailand		SAMN12254043	PRJNA438219	Submitted2019
PV170_2019-5-06_SouthAmerica_Colombia_Cordoba	PV170	clinical	2019-05-06	SouthAmerica	Colombia	Cordoba	SAMN20804964	PRJNA754786	Submitted2021
PV173_2019-7-17_SouthAmerica_Colombia_Cordoba	PV173	NA	2019-07-17	SouthAmerica	Colombia	Cordoba	SAMN20804965	PRJNA754787	Submitted2021
PV278_2019-7-30_SouthAmerica_Colombia_Bogota	PV278	clinical	2019-07-30	SouthAmerica	Colombia	Bogota	SAMN20804967	PRJNA754787	Submitted2021
PV280_2019-7-24_SouthAmerica_Colombia_Cordoba	PV280	clinical	2019-07-24	SouthAmerica	Colombia	Cordoba	SAMN20804968	PRJNA754787	Submitted2021
PV53_2018-7-30_SouthAmerica_Colombia_Cordoba	PV53	clinical	2018-07-30	SouthAmerica	Colombia	Cordoba	SAMN20804969	PRJNA754788	Submitted2021
PV85_2017-10-16_SouthAmerica_Colombia_Cordoba	PV85	clinical	2017-10-16	SouthAmerica	Colombia	Cordoba	SAMN20804970	PRJNA754789	Submitted2021
GCA000182345_1980-7-01_Asia_Japan * pre-pandemic sample	VP161546	clinical	1980	Asia	Japan		SAMEA8103018	PRJEB39490	
GCA000182385_1996-7-01_Asia_Japan_Osaka	RIMD 2210633	NA	1996	Asia	Japan	Osaka	SAMD00058707	PRJNA360	
GCA000182465_1996-7-01_Asia_Thailand	S094	clinical	1996	Asia	Thailand		SAMN02338947	PRJNA215961	
GCA000196095_1996-7-01_Asia_China_Taiwan	S092	clinical	1996	Asia	China	Taiwan	SAMN02338945	PRJNA215961	
GCA000454455_1997-7-01_Asia_China_Taiwan	S074	clinical	1997	Asia	China	Taiwan	SAMN02338931	PRJNA215961	
GCA000489695_1997-7-01_Asia_China_Taiwan	S068	clinical	1997	Asia	China	Taiwan	SAMN02338925	PRJNA215961	
GCA000489715_1997-7-01_Asia_China_Taiwan	S067	clinical	1997	Asia	China	Taiwan	SAMN02338924	PRJNA215961	
GCA000489735_1997-7-01_Asia_China_Taiwan	S066	clinical	1997	Asia	China	Taiwan	SAMN02338923	PRJNA215961	
GCA000489835_1998-7-01_Asia_Bangladesh	AN-5034	NA	1998	Asia	Bangladesh		SAMN02436265	PRJNA33625	
GCA000489915_1998-7-01_Asia_Singapore	S088	clinical	1998	Asia	Singapore		SAMN02338942	PRJNA215961	
GCA000490465_1998-7-01_Asia_Singapore	S087	clinical	1998	Asia	Singapore		SAMN02338941	PRJNA215961	
GCA000490515_1998-7-01_Asia_Japan	S083	clinical	1998	Asia	Japan		SAMN02338939	PRJNA215961	
GCA000490555_1998-7-01_Asia_China_Taiwan	S064	clinical	1998	Asia	China	Taiwan	SAMN02338921	PRJNA215961	
GCA000490595_1998-7-01_Asia_Singapore	S062	clinical	1998	Asia	Singapore		SAMN02338919	PRJNA215961	
GCA000490615_1998-1-01_SouthAmerica_Chile_Antofagasta	ATC220	clinical	1998	SouthAmerica	Chile	Antofagasta	SAMN02781334	PRJNA233509	
GCA000490895_1999-7-01_Asia_India	S091	clinical	1999	Asia	India		SAMN02338944	PRJNA215961	
GCA000490915_1999-7-01_Asia_China_Taiwan	S090	clinical	1999	Asia	China	Taiwan	SAMN02338943	PRJNA215961	

GCA000490935_1999-7-01_Asia_Thailand	S086	clinical	1999	Asia	Thailand		SAMN02338940	PRJNA215961	
GCA000490955_1999-7-01_Asia_China_Taiwan	S078	clinical	1999	Asia	China	Taiwan	SAMN02338935	PRJNA215961	
GCA000490975_1999-7-01_Asia_China_Taiwan	S077	clinical	1999	Asia	China	Taiwan	SAMN02338934	PRJNA215961	
GCA000490995_1999-7-01_Asia_China_Taiwan	S076	clinical	1999	Asia	China	Taiwan	SAMN02338933	PRJNA215961	
GCA000491015_1999-7-01_Asia_China_Taiwan	S075	clinical	1999	Asia	China	Taiwan	SAMN02338932	PRJNA215961	
GCA000500505_1999-7-01_NorthAmerica_Mexico	CICESE-186	clinical	1999	NorthAmerica	Mexico		SAMN13893119	PRJNA602337	
GCA000519365_2000-1-01_SouthAmerica_Peru	2568-00	clinical	2000	SouthAmerica	Peru		SAMN15428945	PRJNA643807	
GCA000521825_2000-1-01_SouthAmerica_Peru	325-00	clinical	2000	SouthAmerica	Peru		SAMN15428940	PRJNA643807	
GCA000522025_2000-1-01_SouthAmerica_Peru	403-00	clinical	2000	SouthAmerica	Peru		SAMN15428941	PRJNA643807	
GCA000522065_2000-1-01_SouthAmerica_Peru	327-00	clinical	2000	SouthAmerica	Peru		SAMN15428942	PRJNA643807	
GCA000524535_2003-7-01_Asia_China_Guangxi	S135	clinical	2003	Asia	China	Guangxi	SAMN02338983	PRJNA215961	
GCA000525005_2000-1-01_SouthAmerica_Peru	2434-00	clinical	2000	SouthAmerica	Peru		SAMN15428944	PRJNA643807	
GCA000558885_2000-1-01_SouthAmerica_Peru	706-00	clinical	2000	SouthAmerica	Peru		SAMN15428939	PRJNA643807	
GCA000571915_2000-1-01_SouthAmerica_Peru	404-00	clinical	2000	SouthAmerica	Peru		SAMN15428938	PRJNA643807	
GCA000707805_2004-7-01_Asia_China_Guangxi	S136	clinical	2004	Asia	China	Guangxi	SAMN02338984	PRJNA215961	
GCA000877475_2004-7-01_NorthAmerica_Canada_Ontario	237865	clinical	2004	NorthAmerica	Canada	Ontario	SAMN03452289	PRJNA275536	
GCA000877485_2004-7-01_NorthAmerica_Canada_Ontario	237135	clinical	2004	NorthAmerica	Canada	Ontario	SAMN03349598	PRJNA275536	
GCA000951795_2004-7-01_NorthAmerica_Canada_BritishColumbia	A4EZ927	clinical	2004	NorthAmerica	Canada	BritishColumbia	SAMN04327443	PRJNA304021	
GCA000972045_2004-1-01_SouthAmerica_Chile_PuertoMontt	PMC48	clinical	2004	SouthAmerica	Chile	PuertoMontt	SAMN02781337	PRJNA233509	
GCA001270815_2004-7-01_NorthAmerica_Mexico	CAIM 1400	clinical	2004	NorthAmerica	Mexico		SAMN13893117	PRJNA602337	
GCA001270825_2005-7-01_Asia_China_Guangxi	S137	clinical	2005	Asia	China	Guangxi	SAMN02338985	PRJNA215961	
GCA001270905_2005-7-01_Asia_China_Liaoning	S133	clinical	2005	Asia	China	Liaoning	SAMN02338981	PRJNA215961	
GCA001270975_2005-7-01_NorthAmerica_Canada_BritishColumbia	A5Z853	clinical	2005	NorthAmerica	Canada	BritishColumbia	SAMN04377388	PRJNA304021	
GCA001608785_2006-7-01_NorthAmerica_USA	605	NA	2006	NorthAmerica	USA		SAMN02597368	PRJNA176634	
GCA001608835_2006-7-01_NorthAmerica_Canada_BritishColumbia	F63267	clinical	2006	NorthAmerica	Canada	BritishColumbia	SAMN04422055	PRJNA304021	
GCA001609525_2007-7-01_Asia_China_Hubei	S138	clinical	2007	Asia	China	Hubei	SAMN02338986	PRJNA215961	
GCA001720365_2007-7-01_NorthAmerica_USA_Washington	863	clinical	2007	NorthAmerica	USA	Washington	SAMN02204307	PRJNA188204	
GCA001895435_2008-7-01_Asia_HongKong	VIP4-0407	clinical	2008	Asia	HongKong		SAMN02471133	PRJNA222557	
GCA001895445_2008-7-01_Asia_Bangladesh	EKP-026	environmental	2008	Asia	Bangladesh		SAMN02641511	PRJNA176652	
GCA001895595_2010-6-28_Asia_China_Guangdong	100151	clinical	2010-06-28	Asia	China	Guangdong	SAMN05935535	PRJNA350230	
GCA001895665_2008-7-01_Asia_China_Shenzhen	VP357	clinical	2008	Asia	China	Shenzhen	SAMN16783253	PRJNA677930	
GCA001895695_2008-7-01_Asia_China_Shenzhen	VP354	clinical	2008	Asia	China	Shenzhen	SAMN16783250	PRJNA677930	
GCA002018625_2008-7-01_Asia_China_Shenzhen	VP137	clinical	2008	Asia	China	Shenzhen	SAMN16783060	PRJNA677930	
GCA003056625_2009-7-01_Asia_China_Shenzhen	VP143	clinical	2009	Asia	China	Shenzhen	SAMN16783066	PRJNA677930	
GCA003056645_2009-7-01_Asia_China_Shenzhen	VP141	clinical	2009	Asia	China	Shenzhen	SAMN16783064	PRJNA677930	
GCA003056675_2017-7-19_Asia_Lebanon	VPF-1	clinical	2017-07-19	Asia	Lebanon		SAMN08667565	PRJNA437553	
GCA003056745_2017-10-18_Asia_Lebanon	VPF-7	clinical	2017-10-18	Asia	Lebanon		SAMN08667669	PRJNA437564	
GCA003057295_2009-7-01_Asia_China_Shenzhen	VP121	clinical	2009	Asia	China	Shenzhen	SAMN16783044	PRJNA677930	
GCA003057315_2009-7-01_Asia_China_Shenzhen	VP96	clinical	2009	Asia	China	Shenzhen	SAMN16783023	PRJNA677930	
GCA003408895_2010-7-01_Asia_China_Shenzhen	VP218	clinical	2010	Asia	China	Shenzhen	SAMN16783127	PRJNA677930	
GCA004006515_2010-1-01_SouthAmerica_Peru	293-10	clinical	2010	SouthAmerica	Peru		SAMN15428966	PRJNA643807	
GCA006368305_2011-7-01_Asia_China_Shenzhen	VP178	clinical	2011	Asia	China	Shenzhen	SAMN16783097	PRJNA677930	
GCA006368345_2011-7-01_Asia_China_Shenzhen	VP176	clinical	2011	Asia	China	Shenzhen	SAMN16783096	PRJNA677930	
GCA006368425_2011-7-01_Asia_China_Shenzhen	VP112	clinical	2011	Asia	China	Shenzhen	SAMN16783036	PRJNA677930	
GCA006368435_2014-7-01_Asia_China_Guangxi	G1_9	environmental_fish	2014	Asia	China	Guangxi	SAMN07338219	PRJNA393608	
GCA006368505_2014-7-01_Asia_China_Guangxi	G1_3	environmental	2014	Asia	China	Guangxi	SAMN07338213	PRJNA393608	
GCA006368595_2012-7-01_Asia_China_Shanghai	VPD14	NA	2012	Asia	China	Shanghai	SAMN09874427	PRJNA487159	
GCA006370625_2014-7-01_Asia_China_Guangxi	F3_9	environmental	2014	Asia	China	Guangxi	SAMN07338153	PRJNA393608	
GCA006370635_2014-7-01_Asia_China_Guangxi	F3_7	environmental_shellfish	2014	Asia	China	Guangxi	SAMN07338151	PRJNA393608	
GCA006370655_2014-7-01_Asia_China_Guangxi	F2_9	environmental	2014	Asia	China	Guangxi	SAMN07338143	PRJNA393608	
GCA006370685_2014-7-01_Asia_China_Guangxi	F3_10	environmental	2014	Asia	China	Guangxi	SAMN07338145	PRJNA393608	
GCA006370925_2014-7-01_Asia_China_Guangxi	F3_1	environmental_fish	2014	Asia	China	Guangxi	SAMN07338144	PRJNA393608	
GCA006371125_2012-7-01_Asia_China_Shenzhen	VP188	clinical	2012	Asia	China	Shenzhen	SAMN16783107	PRJNA677930	
GCA006371195_2012-7-01_Asia_China_Shenzhen	VP170	clinical	2012	Asia	China	Shenzhen	SAMN16783090	PRJNA677930	
GCA006371215_2012-7-01_Asia_China_Shenzhen	VP146	clinical	2012	Asia	China	Shenzhen	SAMN16783069	PRJNA677930	
GCA006371225_2012-7-01_Asia_China_Shenzhen	VP111	clinical	2012	Asia	China	Shenzhen	SAMN16783035	PRJNA677930	
GCA006382955_2013-7-01_Asia_China_Shenzhen	VP65	clinical	2013	Asia	China	Shenzhen	SAMN16783356	PRJNA677930	
GCA009936575_2014-7-01_Asia_China_Guangxi	F3_8	environmental_fish	2014	Asia	China	Guangxi	SAMN07338152	PRJNA393608	
GCA009936605_2014-7-01_Asia_China_Guangxi	F2_7	environmental	2014	Asia	China	Guangxi	SAMN07338141	PRJNA393608	
GCA009991445_2014-7-01_Asia_China_Guangxi	F2_10	environmental_fish	2014	Asia	China	Guangxi	SAMN07338135	PRJNA393608	
GCA009991625_2014-7-01_Asia_China_Guangxi	F2_8	environmental_fish	2014	Asia	China	Guangxi	SAMN07338142	PRJNA393608	
GCA009991675_2014-1-01_SouthAmerica_Peru	G1	clinical	2014	SouthAmerica	Peru		SAMN12364839	PRJNA556706	
GCA010692765_2015-1-01_SouthAmerica_Peru	249-15	clinical	2015	SouthAmerica	Peru		SAMN12364841	PRJNA556706	
GCA015680975_2015-7-01_Asia_China_Shenzhen	VP374	clinical	2015	Asia	China	Shenzhen	SAMN16783270	PRJNA677930	
GCA015784365_2014-7-01_Asia_China_Guangxi	G1_8	environmental_shellfish	2014	Asia	China	Guangxi	SAMN07338218	PRJNA393608	
GCA015789655_2013-7-01_Asia_China_Shenzhen	VP384	clinical	2013	Asia	China	Shenzhen	SAMN16783279	PRJNA677930	
GCA015790155_2011-7-01_Asia_China_Shenzhen	VP185	clinical	2011	Asia	China	Shenzhen	SAMN16783104	PRJNA677930	
GCA015790415_2010-1-01_SouthAmerica_Peru	091-10	clinical	2010	SouthAmerica	Peru		SAMN15428964	PRJNA643807	
GCA015798015_2010-1-01_SouthAmerica_Peru	092-10	clinical	2010	SouthAmerica	Peru		SAMN15428968	PRJNA643807	

GCA015798155_2010-1-01_SouthAmerica_Peru	361-10	clinical	2010	SouthAmerica	Peru		SAMN15428969	PRJNA643807	
GCA015798375_2010-1-01_SouthAmerica_Peru	454-10	clinical	2010	SouthAmerica	Peru		SAMN15428970	PRJNA643807	
GCA015799215_2010-7-01_Asia_China_Shenzhen	VP100	clinical	2010	Asia	China	Shenzhen	SAMN16783025	PRJNA677930	
GCA015803475_2010-7-01_Asia_China_Shenzhen	VP222	clinical	2010	Asia	China	Shenzhen	SAMN16783131	PRJNA677930	
GCA015805555_2009-7-01_Asia_China_Shenzhen	VP89	clinical	2009	Asia	China	Shenzhen	SAMN16783017	PRJNA677930	
GCA015805595_2009-7-01_Asia_China_Shenzhen	VP85	clinical	2009	Asia	China	Shenzhen	SAMN16783013	PRJNA677930	
GCA015805695_2009-7-01_Asia_China_Shenzhen	VP88	clinical	2009	Asia	China	Shenzhen	SAMN16783016	PRJNA677930	
GCA015805755_2009-7-01_Asia_China_Shenzhen	VP87	clinical	2009	Asia	China	Shenzhen	SAMN16783015	PRJNA677930	
GCA015805805_2009-7-01_Asia_China_Shenzhen	VP90	clinical	2009	Asia	China	Shenzhen	SAMN16783018	PRJNA677930	
GCA015805855_2009-7-01_Asia_China_Shenzhen	VP91	clinical	2009	Asia	China	Shenzhen	SAMN16783019	PRJNA677930	
GCA016817415_2015-7-01_Asia_China_Shenzhen	VP302	clinical	2015	Asia	China	Shenzhen	SAMN16783203	PRJNA677930	
GCA016817945_2015-7-01_Asia_China_Shenzhen	VP285	clinical	2015	Asia	China	Shenzhen	SAMN16783186	PRJNA677930	
GCA016818365_2015-1-01_SouthAmerica_Peru_Lima	H11	clinical	2015	SouthAmerica	Peru	Lima	SAMN15428984	PRJNA643807	
GCA016818535_2015-1-01_SouthAmerica_Peru_Lima	G6	clinical	2015	SouthAmerica	Peru	Lima	SAMN15428983	PRJNA643807	
GCA016818595_2012-7-01_Asia_China_Shenzhen	VP230	clinical	2012	Asia	China	Shenzhen	SAMN16783377	PRJNA677930	
GCA016819175_2016-1-01_SouthAmerica_Peru	164-16	clinical	2016	SouthAmerica	Peru		SAMN12364846	PRJNA556706	
GCA016819195_2016-7-01_Asia_China_Shenzhen	VP8	clinical	2016	Asia	China	Shenzhen	SAMN16783345	PRJNA677930	
GCA016819235_2016-7-01_Asia_China_Shenzhen	VP399	clinical	2016	Asia	China	Shenzhen	SAMN16783294	PRJNA677930	
GCA016819245_2016-7-01_Asia_China_Shenzhen	VP398	clinical	2016	Asia	China	Shenzhen	SAMN16783293	PRJNA677930	
GCA016820795_2016-7-01_Asia_China_Shenzhen	VP209	clinical	2016	Asia	China	Shenzhen	SAMN16783118	PRJNA677930	
GCA016820815_2016-1-01_SouthAmerica_Peru_Lima	H12	clinical	2016	SouthAmerica	Peru	Lima	SAMN15429004	PRJNA643807	
GCA016820835_2016-1-01_SouthAmerica_Peru_Lima	G8	clinical	2016	SouthAmerica	Peru	Lima	SAMN15429003	PRJNA643807	
GCA016821515_2016-1-01_SouthAmerica_Peru	165-16	clinical	2016	SouthAmerica	Peru		SAMN15429002	PRJNA643807	
GCA016821535_2012-7-01_Asia_China_Shenzhen	VP404	clinical	2012	Asia	China	Shenzhen	SAMN16783299	PRJNA677930	
GCA016821575_2012-7-01_Asia_China_Shenzhen	VP402	clinical	2012	Asia	China	Shenzhen	SAMN16783297	PRJNA677930	
GCA016821595_2012-7-01_Asia_China_Shenzhen	VP401	clinical	2012	Asia	China	Shenzhen	SAMN16783296	PRJNA677930	
GCA016821635_2016-7-01_Asia_China_Zhenjiang	ICDC-VP01784	clinical	2016	Asia	China	Zhenjiang	SAMN15294466	PRJNA639932	
GCA016821665_2016-7-01_Asia_China_Suqian	ICDC-VP01786	clinical	2016	Asia	China	Suqian	SAMN15294467	PRJNA639932	
GCA016821735_2008-7-01_Asia_China_Shenzhen	VP394	clinical	2008	Asia	China	Shenzhen	SAMN16783289	PRJNA677930	
GCA016821755_2017-1-01_SouthAmerica_Peru	686-17	clinical	2017	SouthAmerica	Peru		SAMN12364847	PRJNA556706	
GCA016821775_2017-1-01_SouthAmerica_Peru	2214-17	clinical	2017	SouthAmerica	Peru		SAMN12364848	PRJNA556706	
GCA016821815_2017-7-01_Asia_China_Guangzhou	r75	environmental	2017	Asia	China	Guangzhou	SAMN14411235	PRJNA613630	
GCA016821935_2017-7-01_Asia_China_Guangzhou	r79-2	environmental	2017	Asia	China	Guangzhou	SAMN14411236	PRJNA613630	
GCA016822115_2017-7-01_Asia_China_Shenzhen	VP407	clinical	2017	Asia	China	Shenzhen	SAMN16783389	PRJNA677930	
GCA016822145_2017-7-01_Asia_China_Shenzhen	VP72	clinical	2017	Asia	China	Shenzhen	SAMN16783358	PRJNA677930	
GCA016822155_2017-7-01_Asia_China_Shenzhen	VP443	clinical	2017	Asia	China	Shenzhen	SAMN16783337	PRJNA677930	
GCA016822215_2017-7-01_Asia_China_Shenzhen	VP441	clinical	2017	Asia	China	Shenzhen	SAMN16783335	PRJNA677930	
GCA016822305_2017-7-01_Asia_China_Shenzhen	VP440	clinical	2017	Asia	China	Shenzhen	SAMN16783334	PRJNA677930	
GCA016822335_2017-7-01_Asia_China_Shenzhen	VP405	clinical	2017	Asia	China	Shenzhen	SAMN16783300	PRJNA677930	
GCA016822455_2017-7-01_Asia_China_Shenzhen	VP343	clinical	2017	Asia	China	Shenzhen	SAMN16783240	PRJNA677930	
GCA016822545_2017-7-01_Asia_China_Shenzhen	VP341	clinical	2017	Asia	China	Shenzhen	SAMN16783238	PRJNA677930	
GCA016822735_2017-7-01_Asia_China_Shenzhen	VP272	clinical	2017	Asia	China	Shenzhen	SAMN16783173	PRJNA677930	
GCA016822755_2017-7-01_Asia_China_Shenzhen	VP204	clinical	2017	Asia	China	Shenzhen	SAMN16783116	PRJNA677930	
GCA016823455_2017-7-01_Asia_China_Nantong	ICDC-VP01802	clinical	2017	Asia	China	Nantong	SAMN15294476	PRJNA639932	
GCA016823785_2017-7-01_Asia_China_Nanjing	ICDC-VP01800	clinical	2017	Asia	China	Nanjing	SAMN15294474	PRJNA639932	
GCA016824045_2017-7-01_Asia_China_Nanjing	ICDC-VP01799	clinical	2017	Asia	China	Nanjing	SAMN15294473	PRJNA639932	
GCA016824195_2017-7-01_Asia_China_Nantong	ICDC-VP01801	clinical	2017	Asia	China	Nantong	SAMN15294475	PRJNA639932	
GCA016824675_2017-7-01_Asia_China_Huaian	ICDC-VP01794	clinical	2017	Asia	China	Huaian	SAMN15294471	PRJNA639932	
GCA016824755_2017-7-01_Asia_China_Nanjing	ICDC-VP01797	clinical	2017	Asia	China	Nanjing	SAMN15294472	PRJNA639932	
GCA016824925_2018-7-01_Asia_China_Guangzhou	r46	environmental	2018	Asia	China	Guangzhou	SAMN14411234	PRJNA613630	
GCA016824975_2018-7-01_Asia_China_Suqian	ICDC-VP01816	clinical	2018	Asia	China	Suqian	SAMN15294486	PRJNA639932	
GCA016825015_2018-7-01_Asia_China_Yangzhou	ICDC-VP01815	clinical	2018	Asia	China	Yangzhou	SAMN15294485	PRJNA639932	
GCA016825035_2018-7-01_Asia_China_Yangzhou	ICDC-VP01814	clinical	2018	Asia	China	Yangzhou	SAMN15294484	PRJNA639932	
GCA016825055_2018-7-01_Asia_China_Suqian	ICDC-VP01813	clinical	2018	Asia	China	Suqian	SAMN15294483	PRJNA639932	
GCA016825085_2018-7-01_Asia_China_Wuxi	ICDC-VP01812	clinical	2018	Asia	China	Wuxi	SAMN15294482	PRJNA639932	
GCA016825135_2018-7-01_Asia_China_Nanjing	ICDC-VP01805	clinical	2018	Asia	China	Nanjing	SAMN15294478	PRJNA639932	
GCA016825915_2007-6-09_Asia_China_Guangxi	Gxw_7004	clinical	2007-06-09	Asia	China	Guangxi	SAMN04349747	PRJNA306401	
GCA016826195_2008-5-08_Asia_China_Guangdong	100138	clinical	2008-05-08	Asia	China	Guangdong	SAMN05935524	PRJNA350230	
GCA016826235_2008-10-12_Asia_China_Guangdong	100150	clinical	2008-10-12	Asia	China	Guangdong	SAMN05935534	PRJNA350230	
GCA016826255_2009-5-19_Asia_China_Guangdong	100153	clinical	2009-05-19	Asia	China	Guangdong	SAMN05935537	PRJNA350230	
GCA016826275_2009-7-01_Asia_China_Shenzhen	VP140	clinical	2009	Asia	China	Shenzhen	SAMN16783063	PRJNA677930	
GCA016826315_2010-7-12_Asia_China_Guangdong	100152	clinical	2010-07-12	Asia	China	Guangdong	SAMN05935536	PRJNA350230	
GCA016826435_2012-8-22_NorthAmerica_USA_Maryland	CFSAN007450	clinical	2012-08-22	NorthAmerica	USA	Maryland	SAMN02741386	PRJNA245882	
GCA016826475_2013-6-08_Asia_China_Guangdong	100145	clinical	2013-06-08	Asia	China	Guangdong	SAMN05935529	PRJNA350230	
GCA016826485_2012-8-24_NorthAmerica_USA_Maryland	CFSAN007451	clinical	2012-08-24	NorthAmerica	USA	Maryland	SAMN02741387	PRJNA245882	
GCA016826555_2016-8-03_Asia_China_Shanghai	VP161407	clinical	2016-08-03	Asia	China	Shanghai	SAMN09742584	PRJNA483379	
GCA016826585_2016-8-12_Asia_China_Shanghai	VP161168	clinical	2016-08-12	Asia	China	Shanghai	SAMN09742583	PRJNA483379	
GCA016826615_2009-7-01_Asia_China_Shenzhen	VP123	clinical	2009	Asia	China	Shenzhen	SAMN16783046	PRJNA677930	
GCA016826635_2017-8-02_Asia_Lebanon	VPF-3	clinical	2017-08-02	Asia	Lebanon		SAMN08667567	PRJNA437555	

GCA016826795_2017-8-02_Asia_Lebanon	VPF-4	clinical	2017-08-02	Asia	Lebanon		SAMN08667585	PRJNA437557	
GCA016826815_2017-8-02_Asia_Lebanon	VPF-2	clinical	2017-08-02	Asia	Lebanon		SAMN08667566	PRJNA437554	
GCA016826895_2017-8-16_Asia_Lebanon	VPF-5	clinical	2017-08-16	Asia	Lebanon		SAMN08667654	PRJNA437561	
GCA016826915_2017-9-18_Asia_Lebanon	VPF-6	clinical	2017-09-18	Asia	Lebanon		SAMN08667664	PRJNA437563	
GCA016826955_2011-7-01_Asia_China_Shenzhen	VP102	clinical	2011	Asia	China	Shenzhen	SAMN16783027	PRJNA677930	
GCA016826995_2019-1-12_Asia_China_Shanghai	SH112	clinical	2019-01-12	Asia	China	Shanghai	SAMN16205401	PRJNA664306	
GCA016827015_2005-1-01_SouthAmerica_Chile	PMA37.5	clinical	2005-01	SouthAmerica	Chile		SAMN05858273	PRJNA345099	
GCA016827115_2020-8-01_Asia_China_Beihai	BH0083	clinical	2020-08	Asia	China	Beihai	SAMN20294544	PRJNA747744	
GCA016878075_2015-1-01_SouthAmerica_Peru	277-15	clinical	2015	SouthAmerica	Peru		SAMN15428982	PRJNA643807	
GCA016878215_2011-1-01_SouthAmerica_Peru	1218-11	clinical	2011	SouthAmerica	Peru		SAMN15428974	PRJNA643807	
GCA019685835_2017-7-01_Asia_China_Wuxi	ICDC-VP01803	clinical	2017	Asia	China	Wuxi	SAMN15294477	PRJNA639932	
GCA019685975_2016-7-01_Asia_China_Nantong	ICDC-VP01791	clinical	2016	Asia	China	Nantong	SAMN15294469	PRJNA639932	
GCA019686045_2016-7-01_Asia_China_Wuxi	ICDC-VP01787	clinical	2016	Asia	China	Wuxi	SAMN15294468	PRJNA639932	
GCA05331775_2016-7-01_Asia_China	VP161168	clinical	2016	Asia	China		SAMEA8103029	PRJEB39490	
SRR10038615_2019-8-12_NorthAmerica_USA_Massachusetts	PNUSAV000950	clinical	2019-08-12	NorthAmerica	USA	Massachusetts	SAMN12661289	PRJNA266293	
SRR10072285_2019-7-02_NorthAmerica_USA_California	PNUSAV000970	clinical	2019-07-02	NorthAmerica	USA	California	SAMN12699060	PRJNA266293	
SRR1057385_2007-5-15_NorthAmerica_USA_Texas	CDC_K5058	clinical	2007-05-15	NorthAmerica	USA	Texas	SAMN02368300	PRJNA273159	
SRR10738130_2004-7-01_NorthAmerica_USA_Florida	MDOH-04-5M732	clinical	2004	NorthAmerica	USA	Florida	SAMN13634001	PRJNA531481	
SRR1118598_2007-10-06_NorthAmerica_USA_Georgia	CDC_K5528	clinical	2007-10-06	NorthAmerica	USA	Georgia	SAMN02368334	PRJNA273159	
SRR1118627_2006-9-16_NorthAmerica_USA_Massachusetts	CDC_K5010W	clinical	2006-09-16	NorthAmerica	USA	Massachusetts	SAMN02368299	PRJNA273159	
SRR1118628_2006-9-16_NorthAmerica_USA_Massachusetts	CDC_K5010G	clinical	2006-09-16	NorthAmerica	USA	Massachusetts	SAMN02368298	PRJNA273159	
SRR1118635_2007-2-24_NorthAmerica_USA_Georgia	CDC_K4775	clinical	2007-02-24	NorthAmerica	USA	Georgia	SAMN02368289	PRJNA273159	
SRR11217960_2020-2-03_NorthAmerica_USA_Connecticut	PNUSAV001182	clinical	2020-02-03	NorthAmerica	USA	Connecticut	SAMN14266007	PRJNA266293	
SRR11342511_2020-2-24_NorthAmerica_USA_Connecticut	PNUSAV001185	clinical	2020-02-24	NorthAmerica	USA	Connecticut	SAMN14396416	PRJNA266293	
SRR11357102_2015-7-01_Asia_China_Guangzhou	L8	environmental	2015	Asia	China	Guangzhou	SAMN14411232	PRJNA613630	
SRR11357113_2015-7-01_Asia_China_Guangzhou	L7	environmental	2015	Asia	China	Guangzhou	SAMN14411231	PRJNA613630	
SRR11357185_2015-7-01_Asia_China_Guangzhou	L3	environmental	2015	Asia	China	Guangzhou	SAMN14411228	PRJNA613630	
SRR11357277_2015-7-01_Asia_China_Guangzhou	L1	environmental	2015	Asia	China	Guangzhou	SAMN14411226	PRJNA613630	
SRR11431277_2011-5-01_Asia_China_Shenzhen	GIMxtfL65-2011.05	clinical	2011-05	Asia	China	Shenzhen	SAMN06163187	PRJNA357986	
SRR11431278_2011-5-01_Asia_China_Shenzhen	GIMxtfL61-2011.05	clinical	2011-05	Asia	China	Shenzhen	SAMN06162268	PRJNA357947	
SRR13023725_2018-7-01_Asia_China_Dalian	DH13	environmental	2018	Asia	China	Dalian	SAMN15889525	PRJNA633360	
SRR13228921_2020-7-01_NorthAmerica_USA	PNUSAV001478	clinical	2020	NorthAmerica	USA		SAMN17041481	PRJNA266293	
SRR14001960_2021-7-01_NorthAmerica_USA	PNUSAV001496	clinical	2021	NorthAmerica	USA		SAMN18356821	PRJNA266293	
SRR14097089_2018-5-14_NorthAmerica_USA_NewJersey	210265	environmental	2018-05-14	NorthAmerica	USA	NewJersey	SAMN18528236	PRJNA706389	
SRR14420882_2021-7-01_NorthAmerica_USA	PNUSAV001538	clinical	2021	NorthAmerica	USA		SAMN19012332	PRJNA266293	
SRR15178395_2021-7-01_NorthAmerica_USA	PNUSAV001670	clinical	2021	NorthAmerica	USA		SAMN20286032	PRJNA266293	
SRR15178408_2021-7-01_NorthAmerica_USA	PNUSAV001650	clinical	2021	NorthAmerica	USA		SAMN20286048	PRJNA266293	
SRR15488696_2021-7-01_NorthAmerica_USA	PNUSAV001904	clinical	2021	NorthAmerica	USA		SAMN20813321	PRJNA266293	
SRR16277960_2021-7-01_NorthAmerica_USA	PNUSAV002128	clinical	2021	NorthAmerica	USA		SAMN22183831	PRJNA266293	
SRR16301694_2021-7-01_NorthAmerica_USA	PNUSAV002025	clinical	2021	NorthAmerica	USA		SAMN22231286	PRJNA266293	
SRR16959411_2021-10-22_NorthAmerica_USA_Minnesota	PNUSAV002346	clinical	2021-10-22	NorthAmerica	USA	Minnesota	SAMN23215746	PRJNA266293	
SRR16989257_2021-7-01_NorthAmerica_USA	PNUSAV002402	clinical	2021	NorthAmerica	USA		SAMN23311458	PRJNA266293	
SRR1815540_2006-10-01_NorthAmerica_USA_NewYork	CDC_K4637W	clinical	2006-10-01	NorthAmerica	USA	NewYork	SAMN03358829	PRJNA273159	
SRR2134448_1997-1-01_SouthAmerica_Peru	CFSAN018757	clinical	1997	SouthAmerica	Peru		SAMN03941065	PRJNA245882	
SRR3624770_2007-7-01_NorthAmerica_USA_Washington	920	environmental_shellfish	2007	NorthAmerica	USA	Washington	SAMN05194682	PRJNA324079	
SRR3624774_2007-7-01_NorthAmerica_USA_Washington	752	environmental	2007	NorthAmerica	USA	Washington	SAMN05194683	PRJNA324080	
SRR3624775_2007-7-01_NorthAmerica_USA_Washington	783	environmental	2007	NorthAmerica	USA	Washington	SAMN05195061	PRJNA324095	
SRR3628261_2007-7-01_NorthAmerica_USA_Washington	658	environmental	2007	NorthAmerica	USA	Washington	SAMN05195070	PRJNA324096	
SRR3628271_2007-7-01_NorthAmerica_USA_Washington	765	environmental	2007	NorthAmerica	USA	Washington	SAMN05195120	PRJNA324105	
SRR3655232_2007-7-01_NorthAmerica_USA_Washington	4635	clinical	2007	NorthAmerica	USA	Washington	SAMN05220835	PRJNA325137	
SRR3655243_2007-7-01_NorthAmerica_USA_Washington	4703	clinical	2007	NorthAmerica	USA	Washington	SAMN05220837	PRJNA325139	
SRR4244665_1996-7-01_Asia_India	FDAARGOS_191	clinical	1996	Asia	India		SAMN04875528	PRJNA231221	
SRR4407781_2016-4-16_Asia_China	L70	NA	2016-04-16	Asia	China		SAMN05890675	PRJNA347505	
SRR5070652_1996-7-01_Asia_India	CFSAN023533	clinical	1996	Asia	India		SAMN06076986	PRJNA245882	
SRR5071092_1998-7-01_NorthAmerica_USA_NewYork	CFSAN023542	clinical	1998	NorthAmerica	USA	NewYork	SAMN06076993	PRJNA245882	
SRR5071093_1998-7-01_Asia_Bangladesh	CFSAN023545	clinical	1998	Asia	Bangladesh		SAMN06076996	PRJNA245882	
SRR5071094_1998-7-01_NorthAmerica_USA_Texas	CFSAN023541	clinical	1998	NorthAmerica	USA	Texas	SAMN06076992	PRJNA245882	
SRR5071095_1998-7-01_NorthAmerica_USA_NewYork	CFSAN023543	clinical	1998	NorthAmerica	USA	NewYork	SAMN06076994	PRJNA245882	
SRR5071096_1998-7-01_NorthAmerica_USA_NewYork	CFSAN023544	clinical	1998	NorthAmerica	USA	NewYork	SAMN06076995	PRJNA245882	
SRR5071097_1996-7-01_Asia_India	CFSAN023536	clinical	1996	Asia	India		SAMN06076988	PRJNA245882	
SRR5071098_1998-7-01_Asia_Bangladesh	CFSAN023538	clinical	1998	Asia	Bangladesh		SAMN06076990	PRJNA245882	
SRR5071099_1997-7-01_Asia_India	CFSAN023537	clinical	1997	Asia	India		SAMN06076989	PRJNA245882	
SRR5071100_1998-7-01_Asia_SouthKorea	CFSAN023540	clinical	1998	Asia	SouthKorea		SAMN06076991	PRJNA245882	
SRR5071101_1996-7-01_Asia_India	CFSAN023535	clinical	1996	Asia	India		SAMN06076987	PRJNA245882	
SRR5071103_1998-7-01_Asia_Bangladesh	CFSAN023548	clinical	1998	Asia	Bangladesh		SAMN06076999	PRJNA245882	
SRR5071105_1999-7-01_Asia_Thailand	CFSAN023547	clinical	1999	Asia	Thailand		SAMN06076998	PRJNA245882	
SRR5071106_1999-7-01_Asia_Bangladesh	CFSAN023546	clinical	1999	Asia	Bangladesh		SAMN06076997	PRJNA245882	
SRR5071138_2005-1-01_SouthAmerica_Chile	CFSAN023556	environmental	2005	SouthAmerica	Chile		SAMN06077007	PRJNA245882	
SRR5071140_1998-7-01_Asia_Japan	CFSAN023564	clinical	1998	Asia	Japan		SAMN06077015	PRJNA245882	

SRR5071143_1999-7-01_Asia_Thailand	CFSAN023563	clinical	1999	Asia	Thailand		SAMN06077014	PRJNA245882	
SRR5071144_2000-7-01_Asia_Bangladesh	CFSAN023566	clinical	2000	Asia	Bangladesh		SAMN06077017	PRJNA245882	
SRR5810292_2017-6-09_NorthAmerica_USA_Massachusetts	PNUSAV000061	clinical	2017-06-09	NorthAmerica	USA	Massachusetts	SAMN07327346	PRJNA266293	
SRR5810302_2017-5-15_NorthAmerica_USA_Massachusetts	PNUSAV000060	clinical	2017-05-15	NorthAmerica	USA	Massachusetts	SAMN07327345	PRJNA266293	
SRR5990961_2017-4-29_NorthAmerica_USA_California	PNUSAV000055	clinical	2017-04-29	NorthAmerica	USA	California	SAMN07559594	PRJNA266293	
SRR6234963_2017-8-22_NorthAmerica_USA_Maryland	PNUSAV000101	clinical	2017-08-22	NorthAmerica	USA	Maryland	SAMN07838949	PRJNA266293	
SRR6337242_2017-9-08_NorthAmerica_USA_Pennsylvania	PNUSAV000140	clinical	2017-09-08	NorthAmerica	USA	Pennsylvania	SAMN08113868	PRJNA266293	
SRR6410787_2009-1-01_SouthAmerica_Peru	CFSAN029655	environmental	2009	SouthAmerica	Peru		SAMN08225457	PRJNA245882	
SRR6410789_2009-1-02_SouthAmerica_Peru	CFSAN029657	environmental	2009	SouthAmerica	Peru		SAMN08225459	PRJNA245882	
SRR6410790_2009-1-03_SouthAmerica_Peru	CFSAN029652	environmental	2009	SouthAmerica	Peru		SAMN08225458	PRJNA245882	
SRR6476834_2014-7-01_NorthAmerica_USA	CFSAN026732	clinical	2014	NorthAmerica	USA		SAMN08370015	PRJNA245882	
SRR6794364_2018-1-22_NorthAmerica_USA_California	PNUSAV000173	clinical	2018-01-22	NorthAmerica	USA	California	SAMN08627210	PRJNA266293	
SRR6794365_2018-2-03_NorthAmerica_USA_Alaska	PNUSAV000174	clinical	2018-02-03	NorthAmerica	USA	Alaska	SAMN08627207	PRJNA266293	
SRR7232584_2018-1-01_Asia_Malaysia	533448	clinical_travel	2018	Asia	Malaysia		SAMN09280070	PRJNA438219	
SRR7232589_2017-7-01_Asia_Thailand	524167	clinical_travel	2017	Asia	Thailand		SAMN09280067	PRJNA438219	
SRR7232610_2017-7-01_NorthAmerica_USVirginIslands	511793	clinical_travel	2017	NorthAmerica	USVirginIslands		SAMN09280063	PRJNA438219	
SRR7429932_2018-5-21_NorthAmerica_USA_Maryland	PNUSAV000189	clinical	2018-05-21	NorthAmerica	USA	Maryland	SAMN09488306	PRJNA266293	
SRR7532445_2018-7-05_NorthAmerica_USA_Maryland	PNUSAV000202	clinical	2018-07-05	NorthAmerica	USA	Maryland	SAMN09671577	PRJNA266293	
SRR7532460_2018-7-02_NorthAmerica_USA_Maryland	PNUSAV000207	clinical	2018-07-02	NorthAmerica	USA	Maryland	SAMN09671587	PRJNA266293	
SRR7536467_2018-4-18_NorthAmerica_USA_Pennsylvania	PNUSAV000186	clinical	2018-04-18	NorthAmerica	USA	Pennsylvania	SAMN09689475	PRJNA266293	
SRR7551164_2018-7-02_NorthAmerica_USA_Maryland	PNUSAV000223	clinical	2018-07-02	NorthAmerica	USA	Maryland	SAMN09703857	PRJNA266293	
SRR7610444_2018-7-06_NorthAmerica_USA_Virginia	PNUSAV000211	clinical	2018-07-06	NorthAmerica	USA	Virginia	SAMN09726939	PRJNA266293	
SRR7628881_2018-7-14_NorthAmerica_USA_Oregon	PNUSAV000255	clinical	2018-07-14	NorthAmerica	USA	Oregon	SAMN09745133	PRJNA266293	
SRR7757725_2018-7-03_NorthAmerica_USA_Maryland	PNUSAV000356	clinical	2018-07-03	NorthAmerica	USA	Maryland	SAMN09917443	PRJNA266293	
SRR7818137_2018-8-08_NorthAmerica_USA_Oregon	PNUSAV000398	clinical	2018-08-08	NorthAmerica	USA	Oregon	SAMN10033026	PRJNA266293	
SRR7881541_2018-5-12_NorthAmerica_USA_Texas	PNUSAV000438	clinical	2018-05-12	NorthAmerica	USA	Texas	SAMN10095497	PRJNA266293	
SRR7881543_2018-5-30_NorthAmerica_USA_Texas	PNUSAV000439	clinical	2018-05-30	NorthAmerica	USA	Texas	SAMN10095496	PRJNA266293	
SRR7881560_2018-7-31_NorthAmerica_USA_Texas	PNUSAV000446	clinical	2018-07-31	NorthAmerica	USA	Texas	SAMN10095500	PRJNA266293	
SRR7881561_2018-8-12_NorthAmerica_USA_Texas	PNUSAV000447	clinical	2018-08-12	NorthAmerica	USA	Texas	SAMN10095499	PRJNA266293	
SRR7881562_2018-7-19_NorthAmerica_USA_Texas	PNUSAV000444	clinical	2018-07-19	NorthAmerica	USA	Texas	SAMN10095502	PRJNA266293	
SRR7881563_2018-6-29_NorthAmerica_USA_Florida	PNUSAV000442	clinical	2018-06-29	NorthAmerica	USA	Florida	SAMN10095504	PRJNA266293	
SRR7881566_2018-7-17_NorthAmerica_USA_Texas	PNUSAV000445	clinical	2018-07-17	NorthAmerica	USA	Texas	SAMN10095501	PRJNA266293	
SRR7949717_2018-4-07_NorthAmerica_USA_Pennsylvania	PNUSAV000479	clinical	2018-04-07	NorthAmerica	USA	Pennsylvania	SAMN10163151	PRJNA266293	
SRR7949720_2018-7-19_NorthAmerica_USA_Delaware	PNUSAV000480	clinical	2018-07-19	NorthAmerica	USA	Delaware	SAMN10163150	PRJNA266293	
SRR7985075_2018-8-28_NorthAmerica_USA_Ohio	PNUSAV000481	clinical	2018-08-28	NorthAmerica	USA	Ohio	SAMN10221805	PRJNA266293	
SRR8170043_2018-10-07_NorthAmerica_USA_NewYork	PNUSAV000523	clinical	2018-10-07	NorthAmerica	USA	NewYork	SAMN10391026	PRJNA266293	
SRR8187073_2018-10-14_NorthAmerica_USA_NewYork	PNUSAV000525	clinical	2018-10-14	NorthAmerica	USA	NewYork	SAMN10419344	PRJNA266293	
SRR8478913_2018-12-05_NorthAmerica_USA_NorthDakota	PNUSAV000566	clinical	2018-12-05	NorthAmerica	USA	NorthDakota	SAMN10794262	PRJNA266293	
SRR8742512_2019-1-20_NorthAmerica_USA_RhodeIsland	PNUSAV000580	clinical	2019-01-20	NorthAmerica	USA	RhodeIsland	SAMN11159227	PRJNA266293	
SRR9641511_2018-6-18_NorthAmerica_USA_SouthCarolina	PNUSAV000253	clinical	2018-06-18	NorthAmerica	USA	SouthCarolina	SAMN12212336	PRJNA266293	

Table S2. Phylogenetic signals of geography at continent and country level, with p-values based on a two-sided test of variance of phylogenetically independent contrasts (PICs) relative to a phylogeny with shuffled tips

Trait	K stat	PIC.variance.P
continent	0.2441652	0.001
country	0.1764592	0.001

Table S3. Within-population genetic diversity (Hedrick's GST) per cluster, over time

Temporal Period	LatAm-VpST3	Asian Dominant Group	Modern Group
1995-1999	0.008	0.024	
2000-2005	0.013	0.032	0.022
2006-2010	0.016	0.055	0.024
2011-2015	0.041	0.04	0.027
2016-2020		0.045	0.029

Table S4. Mutation and recombination rates for the whole VpST3 population and each cluster

Metric	Whole VpST3 Population	LatAm-VpST3	Asian Dominant Cluster	Modern Cluster
Mutation rate	4.64-5.50x10^{-4}	3.44x10^{-4}	3.66x10^{-4}	5.22x10^{-4}
Recombination rate (r/m ratio)	1.621	0.164	1.235	2.401

Table S5. Comparison of 3 clusters identified by discriminatory analysis of principle components, and the statistically significant clusters found previously, with 98% support confirming the LatAm-VpST3 group (Cluster 1).

TreeStructure Cluster	Average DAPC Posterior for Cluster 1	Average DAPC Posterior for Cluster 2	Average DAPC Posterior for Cluster 3
1	0.982116596	0.017558894	0.00032451
2	0.003282504	0.987662584	0.009054912
3	0.000195191	0.017822141	0.981982668

Table S6. Total Gene Count Statistics

Group	Average number	Max number of genes	Min number of genes	Range of genes
LatAm-VpST3	4573	4735	4402	333
Rest of collection	4551	4729	4292	437

Table S7. LatAm-VpST3 accessory gene presence associated with sea surface temperature anomalies. Associations scored using a two-sided Fisher's exact test in Scoary (68), accounting for population structure using a post-hoc permutation test based on a pairwise comparisons algorithm.

Association	Genes	Notable genes
Specific to positive anomalies (specificity=1.0). Gene only found in positive anomalies (though not always present in positive anomalies).	73	type VI secretion system baseplate subunit TssF<br>DNA repair protein RadC<br>DNA-binding transcriptional regulator CytR<br>type I pantothenate kinase<br>glycosyltransferase<br>inovirus Gp2 family protein<br>MaoC family dehydratase<br>DegT/DnrJ/EryC1/StrS family aminotransferase<br>serine acetyltransferase<br>WYL domain-containing protein<br>glucose-1-phosphate thymidylyltransferase RfbA<br>porin family protein<br>polysaccharide biosynthesis protein<br>N-acetyltransferase<br>1,4-dihydroxy-2-naphthoate polyprenyltransferase<br>capsule biosynthesis GfcC family protein<br>SAM-dependent methyltransferase<br>DNA topoisomerase<br>YjbF family lipoprotein<br>site-specific integrase<br>DNA cytosine methyltransferase<br>TIGR03756 family integrating conjugative element protein<br>ParB/RepB/Spo0J family partition protein<br>NAD-dependent epimerase/dehydratase family protein<br>sugar transferase<br>Y-family DNA polymerase

		hypothetical protein<br><br>FdtA/QdtA family cupin domain-containing protein<br><br>lipid IV(A) 3-deoxy-D-manno-octulosonic acid transferase<br><br>nucleotide sugar dehydrogenase
Sensitive to positive anomalies<br><br>(sensitivity=1.0). Gene present in all positive anomalies (but also present in some negative anomalies).	1	hypothetical protein only
Specific to negative anomalies<br><br>(specificity=1.0). Gene only found in negative anomalies (though not always present in negative anomalies).	0	
Sensitive to negative anomalies<br><br>(sensitivity=1.0). Gene present in all negative anomalies (but also present in some positive anomalies).	5	1,4-dihydroxy-2-naphthoate polyprenyltransferase<br><br>HlyD family type I secretion periplasmic adaptor subunit<br><br>DNA-binding transcriptional regulator CytR<br><br>HslU--HslV peptidase ATPase subunit<br><br>type I pantothenate kinase coaA

Table S8. Sites within genes of interest identified to be under significant pervasive selection by HyPhy SLAC (70)- significance is calculated for each site using an extended binomial distribution hypothesis test

Gene	Annotation	Number of sites under positive selection	Number of sites under negative selection	*p*-value
**Discriminatory SNPs**				
mnmE	tRNA uridine-5-carboxymethylaminomethyl(34) synthesis GTPase	7	3	0.01
**Significant associations with LatAm-VpST3**				
aguA	agmatine deiminase	0	10	0.05
aguB	N-carbamoylputrescine amidase	0	4	0.01
nhaD	sodium:proton antiporter	0	5	0.01
	glutamine synthetase family protein	0	1	0.05
	FAD-binding oxidoreductase	0	5	0.01
	gamma-glutamyl-gamma-aminobutyrate hydrolase family protein	0	2	0.05
	N-6 DNA methylase	14	0	0.01
**Significant associations with SST anomalies**				
radC	DNA repair protein	0	3	0.05
cytR	DNA-binding transcriptional regulator	0	7	0.05
glgC_1	glucose-1-phosphate	0	4	0.01
glgC_2	glucose-1-phosphate	0	2	0.01
	thymidylyltransferase RfbA	4	3	0.01
	porin family protein	0	1	0.01
	porin family protein	0	10	0.05
	porin family protein	0	1	0.01
	capsule biosynthesis GfcC family protein	0	3	0.01
	SAM-dependent methyltransferase	0	2	0.05
	NAD-dependent epimerase/dehydratase family	0	4	0.01
	Y-family DNA polymerase	0	10	0.01
	nucleotide sugar dehydrogenase	0	2	0.05
