# Supplementary material for: Evolutionary dynamics of the successful expansion of pandemic Vibrio parahaemolyticus ST3 in Latin America
Source: Nat Commun. 2024 Sep 7;15:7828. doi: 10.1038/s41467-024-52159-y (PMC11380683; doi:10.1038/s41467-024-52159-y)
Supplement: Supplementary file 3 — Reporting Summary [file 41467_2024_52159_MOESM3_ESM.pdf]

nature portfolio

Corresponding author(s): J. Martinez-Urtaza
Last updated by author(s): Aug 12, 2024

Reporting Summary

Nature Portfolio wishes to improve the reproducibility of the work that we publish. This form provides structure for consistency and transparency in reporting. For further information on Nature Portfolio policies, see our Editorial Policies and the Editorial Policy Checklist.

Statistics

For all statistical analyses, confirm that the following items are present in the figure legend, table legend, main text, or Methods section.

n/a	Confirmed	
☒	☐	The exact sample size (*n*) for each experimental group/condition, given as a discrete number and unit of measurement
☒	☐	A statement on whether measurements were taken from distinct samples or whether the same sample was measured repeatedly
☐	☒	The statistical test(s) used AND whether they are one- or two-sided<br>*Only common tests should be described solely by name; describe more complex techniques in the Methods section.*
☒	☐	A description of all covariates tested
☒	☐	A description of any assumptions or corrections, such as tests of normality and adjustment for multiple comparisons
☒	☐	A full description of the statistical parameters including central tendency (e.g. means) or other basic estimates (e.g. regression coefficient) AND variation (e.g. standard deviation) or associated estimates of uncertainty (e.g. confidence intervals)
☐	☒	For null hypothesis testing, the test statistic (e.g. *F*, *t*, *r*) with confidence intervals, effect sizes, degrees of freedom and *P* value noted<br>*Give P values as exact values whenever suitable.*
☐	☒	For Bayesian analysis, information on the choice of priors and Markov chain Monte Carlo settings
☒	☐	For hierarchical and complex designs, identification of the appropriate level for tests and full reporting of outcomes
☒	☐	Estimates of effect sizes (e.g. Cohen's *d*, Pearson's *r*), indicating how they were calculated

Our web collection on statistics for biologists contains articles on many of the points above.

Software and code

Policy information about availability of computer code

Data collection	No software was used for data collection
Data analysis	Sequences were assembled and annotated using Bactopia v2.0.2 and were confirmed as ST3 using MLST v2.11. A core genome alignment was created using parsnp v1.5.6 with recombination removed using Gubbins v3.1.6. SNPs were obtained using snp-sites v2.5.1 and annotated for predicted functional effect using SnpEff v5.1. IQ-TREE v2.2.3 was used to construct maximum likelihood phylogenetic tree. TempEst v1.5.3 confirmed a temporal signal in the tree. BEAST v2 was used for Bayesian phylogeographic analysis. Picante v1.8.2 tested for a geographical signal in the phylogenetic tree while TreeStructure v0.1.0 identified significant groups within the tree. Pangenome was determined using Roary v3.13.0, with statistical association analysis of accessory genes performed using Scoary v1.6.16. Site-specific selection pressures were estimated using Single-Likelihood Ancestor Counting (SLAC) within HyPhy v2.5.48. R packages used included adegenet v2.1.10 for Discriminant Analysis of Principle Components, vcfR v1.15, popR v2.6.1, dartR v2.9.7, vegan 2.6.4. Remote sensing climate data were processed and zonally extracted using xarray in Python.

For manuscripts utilizing custom algorithms or software that are central to the research but not yet described in published literature, software must be made available to editors and reviewers. We strongly encourage code deposition in a community repository (e.g. GitHub). See the Nature Portfolio guidelines for submitting code & software for further information.

Data

Policy information about availability of data

All manuscripts must include a data availability statement. This statement should provide the following information, where applicable:

- Accession codes, unique identifiers, or web links for publicly available datasets
- A description of any restrictions on data availability
- For clinical datasets or third party data, please ensure that the statement adheres to our policy

The sequence data generated in this study have been deposited in a public repository on NCBI within BioProject number PRJNA1062747. The metadata and individual accession numbers for these sequences can be found in Appendix Table 1.
Sequence of the new genomes included in this study can be accessed online https://figshare.com/s/73c3b26a5fe16b2876cf

Research involving human participants, their data, or biological material

Policy information about studies with human participants or human data. See also policy information about sex, gender (identity/presentation), and sexual orientation and race, ethnicity and racism.

Reporting on sex and gender	NA
Reporting on race, ethnicity, or other socially relevant groupings	NA
Population characteristics	NA
Recruitment	NA
Ethics oversight	Strains were submitted from regional laboratories to the reference center at the Instituto Nacional de Salud (Lima, Peru) and only information about the location and date of isolation was provided.<br><br>This study was conducted within the framework of the National surveillance for Acute Enteric Diarrhea approved by the Instituto Nacional de Salud of Peru and the Committee of Research and Ethics approval was waived in accordance with the national legislation and the institutional requirements for Public Health Surveillance (Ministry Resolution N.° 730-2022-MINSA).

Note that full information on the approval of the study protocol must also be provided in the manuscript.

Field-specific reporting

Please select the one below that is the best fit for your research. If you are not sure, read the appropriate sections before making your selection.

☐ Life sciences ☐ Behavioural & social sciences ☒ Ecological, evolutionary & environmental sciences

For a reference copy of the document with all sections, see nature.com/documents/nr-reporting-summary-flat.pdf

Ecological, evolutionary & environmental sciences study design

All studies must disclose on these points even when the disclosure is negative.

Study description	Using a global collection of clinical and environmental VpST3 isolates, including novel Latin American isolates, we reconstructed the phylogenetic history of VpST3 and tested for population structure. The pangenome was constructed to annotate the presence of accessory genes within the collection. Oceanic climate data was acquired to facilitate the identification of adaptation signatures, using evolutionary and statistical association analysis.
Research sample	434 publicly-available VpST3 genomes (narrowed down to 280) and 32 novel VpST3 genomes
Sampling strategy	We used all sequences submitted from regional labs to the reference lab at the Instituto Nacional de Salud of Peru for the novel sequences. Publicly-accessible strains were selected for analysis from global databases based on two criteria; the samples were accompanied with sufficient metadata (minimum of a year and country) and the samples were confirmed as ST3 using multi-locus sequence typing based on a series of genetic markers, in MLST v2.11.
Data collection	A total of 32 novel VpST3 strains from the National Centre for Public Health in Peru, collected between 1997-2007 from regions spanning Iquitos in Northern Peru, to Puerto Montt in southern Chile, were sequenced using MiSeq Illumina. Pre-existing sequences were identified and downloaded using NCBI search utility functions. Oceanic climate data was acquired from publicly accessible data repositories for the period of interest.

Timing and spatial scale	Our study was global, with a full temporal range of 1996-2021 (with the addition of a 'pre-pandemic' isolate from 1980 used as an outgroup for rooting).
Data exclusions	Reads that failed to pass length or quality requirements (minimum 1kb read length and average quality Q10) were filtered out and excluded from downstream analyses. We excluded the limited number of strains found outside our 3 continents of interest (Asia, North America, South America). Of the 434 publicly available VpST3 genomes, 154 were excluded.
Reproducibility	All accession numbers for sequences, software and environmental data are publicly and freely available for replication . Parameters are listed in methodology.
Randomization	Choice of genomes was not random- it was based on sufficient metadata and quality of sequences.
Blinding	Novel strains were anonymised.

Did the study involve field work? ☐ Yes ☒ No

Reporting for specific materials, systems and methods

We require information from authors about some types of materials, experimental systems and methods used in many studies. Here, indicate whether each material, system or method listed is relevant to your study. If you are not sure if a list item applies to your research, read the appropriate section before selecting a response.

Materials & experimental systems

n/a	Involved in the study
☒	☐ Antibodies
☒	☐ Eukaryotic cell lines
☒	☐ Palaeontology and archaeology
☒	☐ Animals and other organisms
☒	☐ Clinical data
☒	☐ Dual use research of concern
☒	☐ Plants

Methods

n/a	Involved in the study
☒	☐ ChIP-seq
☒	☐ Flow cytometry
☒	☐ MRI-based neuroimaging

Plants

Seed stocks	*Report on the source of all seed stocks or other plant material used. If applicable, state the seed stock centre and catalogue number. If plant specimens were collected from the field, describe the collection location, date and sampling procedures.*
Novel plant genotypes	*Describe the methods by which all novel plant genotypes were produced. This includes those generated by transgenic approaches, gene editing, chemical/radiation-based mutagenesis and hybridization. For transgenic lines, describe the transformation method, the number of independent lines analyzed and the generation upon which experiments were performed. For gene-edited lines, describe the editor used, the endogenous sequence targeted for editing, the targeting guide RNA sequence (if applicable) and how the editor was applied.*
Authentication	*Describe any authentication procedures for each seed stock used or novel genotype generated. Describe any experiments used to assess the effect of a mutation and, where applicable, how potential secondary effects (e.g. second site T-DNA insertions, mosiacism, off-target gene editing) were examined.*
